# Contamination of *Streptococcus suis* in pork and edible pig organs in central Thailand

**DOI:** 10.14202/vetworld.2019.165-169

**Published:** 2019-01-29

**Authors:** Nuchjaree Boonyong, Sarawan Kaewmongkol, Duangdaow Khunbutsri, Khomsan Satchasataporn, Nattakan Meekhanon

**Affiliations:** Department of Veterinary Technology, Faculty of Veterinary Technology, Kasetsart University, Bangkok 10900, Thailand

**Keywords:** contamination, loop-mediated isothermal amplification, pork, *Streptococcus suis*, Thailand

## Abstract

**Background and Aim:**

*Streptococcus suis* is an important zoonotic pathogen that can cause serious diseases in both swine and humans worldwide, especially in Asian countries. Since the majority of human cases reported in Thailand were infected by the consumption of a raw pork dish, the microbial food safety hazard associated with raw meat has been a matter of concern. Therefore, this study aimed to investigate the contamination by *S. suis* in pork and edible pig organs sold in central Thailand.

**Materials and Methods:**

In total, 88 raw pork and pig organ samples were purchased from markets, butcher shops, and supermarkets in central Thailand. The samples were examined using the loop-mediated isothermal amplification (LAMP) technique. LAMP reactions used for the detection of the DNA of *S. suis* (LAMP_SS_) and *S. suis* serotype 2 or 1/2 (LAMP_SS2_) were carried out according to previous studies.

**Results:**

The percentage of LAMP_SS_-positive samples was as high as 85.23% (75/88) while the percentage of LAMP_SS2_-positive samples was 17.05% (15/88). The percentages of LAMP_SS_- and LAMP_SS2_-positive samples were relatively high in both pig organs (lung and heart) and meat (sliced pork and minced pork) compared with the previous report. Except one supermarket, LAMP_SS_-positive samples were found in all sources investigated in this study. The pork and pig organs obtained from the markets and the butcher shops additionally gave positive results for LAMP_SS2_.

**Conclusion:**

Using LAMP techniques, high rate contamination of *S. suis* was found in raw pork and edible pig organs sold at different sources in central Thailand. The cross-contamination could have occurred through slaughtering, meat cutting, and meat handling processes. Therefore, consumers and people involved in the pig production industry should be aware of the potential hazards of *S. suis* infection; food safety education is crucial to prevent further infection.

## Introduction

*Streptococcus suis* is a well-recognized zoonotic pathogen worldwide. *S. suis* infection can cause serious diseases in swine as well as severe consequences in humans. The most striking clinical feature found in infected patients is meningitis, and after recovery, the patients frequently suffer from permanent loss of hearing [1]. Other clinical manifestations including arthritis, endocarditis, pneumonia, septicemia, and septic shock have been reported in human infections [1]. Since the first human *S. suis* infection was reported in Denmark in 1968, >1600 human cases have occurred in many countries, particularly in Vietnam, Thailand, and China [2]. Although direct contact through a wound is the main transmission route of human infection, the majority of human cases reported in Vietnam and Thailand were infected by the consumption of raw pork and offal [3,4].

Based on the various capsular polysaccharide (CPS) antigens, *S. suis* strains have been classified into different serotypes [2]. Of the serotypes described so far, *S. suis* serotype 2 is the most prevalent and pathogenic for both pigs and humans [5,6]. In addition, other serotypes including serotypes 1, 4, 5, 9, 14, 16, 21, 24, and 31 have been occasionally reported in human cases [2,7-12]. Since *S. suis* serotype 2 and the other potential hazardous strains could be found even in the slaughtered pigs [13,14], contamination by *S. suis* in pork and pig organs should be taken into account as it could lead to a high risk of human infection. Loop-mediated isothermal amplification (LAMP) specific to *S. suis*, which was previously described elsewhere [15], was used to detect *S. suis* in this study. Since LAMP is simple, fast, and sensitive, this technique can be used as a surveillance tool for the detection of *S. suis* contamination. Accordingly, LAMP techniques targeting the gene-encoding recombination/repair protein (*recN*) and the CPS synthesis gene specific for serotype 2 and 1/2 (*cps2J*) can be used for the detection of *S. suis* and *S. suis* serotype 2 or 1/2, respectively [15,16].

Central Thailand is not only the center of the country’s economy, transportation, and tourism but is also a region where the pig density is high. Therefore, in this study, we aimed to investigate *S. suis* contamination in pork and edible pig organs sold in retail markets, butcher shops, and supermarkets in central Thailand. The results from this study will provide important insights into the risk management of this emerging zoonotic pathogen in food.

## Materials and Methods

### Ethical approval

As live animals were not used, ethical approval for animal research was not required in this study. Pork and pig organ samples were purchased from local markets, butcher shops, and supermarkets in central Thailand.

### Sample collection and preparation

In total, 88 samples, consisting of 50 raw pork samples (27 sliced pork and 23 minced pork) and 38 pig organ samples (16 livers, 13 lungs, and 9 hearts), were purchased from 20 sources (eight markets, eight butcher shops, and four supermarkets) in central Thailand between July 2016 and December 2017. The samples were immediately transported to the Faculty of Veterinary Technology, Kasetsart University, Bangkok, Thailand, and were prepared for *S. suis* detection following a previous study [15] with slight modifications. Briefly, each sample was aseptically cut into small pieces, and 100 g of each sample was subsequently homogenized with the appropriate amount (20-100 ml) of 0.9% saline solution in a sterile stomacher bag for 2 min at 230 rpm using a Stomacher^®^ 400 circulator (Scientific Promotion Co., Ltd., UK). Then, 100 µl of the solution obtained from homogenization was added to 5 ml of Todd-Hewitt broth supplemented with Streptococcus Selective Supplement (Oxoid, UK). After incubation at 37°C for 18-24 h, 1 ml of each bacterial culture was used for DNA extraction.

### Molecular techniques

#### DNA extraction

The genomic DNA was extracted using InstaGene Matrix^®^ (Bio-Rad Laboratories, Inc., United States) according to the manufacturer’s recommendations. All genomic DNA samples were stored at −20°C until used.

#### LAMP

LAMP reactions used for the detection of DNA of *S. suis* (LAMP_SS_) and *S. suis* serotype 2 or 1/2 (LAMP_SS2_) were carried out according to Arai *et al*. [15] and Zhang *et al*. [16], respectively. The primer sets are shown in Table-1 [15,16] and consisted of two inner primers (forward inner primer [FIP] and backward inner primer [BIP]), two outer primers (F3 and B3), and an LB loop primer. The primers in each reaction mixture contained 0.2 µM each of F3 and B3, 1.6 µM each of FIP and BIP, and 0.8 µM of LB loop primer. LAMP reactions were performed using a Loopamp DNA amplification kit (Eiken Chemical Co., Ltd., Japan) using the GeneAmp^®^ PCR system 9700 (Applied Biosystem, Singapore). LAMP_SS_ reaction was carried out at 60°C for 60 min followed by inactivation at 80°C for 5 min. The samples which gave a positive result for LAMP_SS_ were further examined using LAMP_SS2_. LAMP_SS2_ was performed at 63°C for 60 min and was then inactivated at 85°C for 2 min. In all reactions, *S. suis* strain P1/7 [17] and distilled water were used as positive and negative controls, respectively.

**Table-1:** Primer sequences of LAMP_SS_ and LAMP_SS2_ used in this study.

Assay	Primer	Primer sequence (5’–3’)	References
LAMP_SS_	F3	TGTCGATGATGTTTTGGACTA	[15]
	B3	GCTTTCTCCATATACAAGTCTTG	
	FIP	AAGCTGAACTTCCAAATCATCTCCCAGCGAAGAATACAATCTATTGAC	
	BIP	TAGAGAAAGAATTGGTTGAACGAGCGCGGATAATATCTTCTAAAACAAC	
	LB	GGTCAGCTCAGCCAATCACGC	
LAMP_SS2_	F3	GTGTTTCAAACGAAGGAAT	[16]
	B3	GCACCTCTTTTATCTCTTCCAA	
	FIP	GTTGCCGTCAACAATATCATCAGAACGGTATCAAAAATAGCACAGC	
	BIP	AGAGAATGATAGTGATTTGTCGGG TTTGCAGCTCAGATTCTTG	
	LB	AGGGTTACTTGCTACTTTTGATGG	

LAMP=Loop-mediated isothermal amplification, BIP=Backward inner primer, FIP=Forward inner primer

#### LAMP product detection

**Figure-1:**
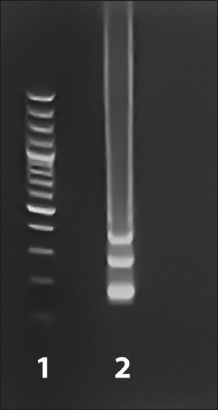
Agarose gel electrophoresis under LED illuminator used to observe the band of loop-mediated isothermal amplification (LAMP) products, where a positive result of LAMP shows a ladder-like band pattern. Lane 1, 100 bp Plus DNA Ladder; Lane 2, LAMP-positive sample.

Gel electrophoresis was used to analyze the products of both LAMP_SS_ and LAMP_SS2_ by HE-Plus Electrophoresis (Hoefer, U.S.A). An UltraSlim^®^ LED illuminator (Maestrogen, Taiwan) was used to observe the bands of products, and the samples were considered to be positive if they showed a ladder-like band pattern (Figure-1).

## Results

All sample types investigated in this study gave positive results with both LAMP_SS_ and LAMP_SS2_ analyses, as shown in Table-2. The percentage of LAMP_SS_-positive samples was as high as 85.23% (75/88) while the percentage of LAMP_SS2_-positive sample was 17.05% (15/88). The highest percentage of LAMP_SS_-positive samples was in the lung (92.31%), followed by the heart (88.89%) and sliced pork (88.89%), while the lowest percentage of LAMP_SS_-positive samples was in the liver. Similar to LAMP_SS_, the results from LAMP_SS2_ analysis showed that the percentage of LAMP_SS2_-positive sample was highest in the lung (30.77%) followed by the heart (22.22%) and sliced pork (18.52%) (Table-2).

**Table-2:** Meat samples used in this study and LAMP results.

Sample type	Number of samples	Number of LAMP_SS_-positive samples (%)	Number of LAMP_SS2_-positive samples (%)
Sliced pork	27	24 (88.89)	5 (18.52)
Minced pork	23	20 (86.96)	4 (17.39)
Liver	16	11 (68.75)	0 (0)
Lung	13	12 (92.31)	4 (30.77)
Heart	9	8 (88.89)	2 (22.22)
Total	88	75 (85.23)	15 (17.05)

LAMP=Loop-mediated isothermal amplification

The results of LAMP analyses classified by the type of sample source are shown in Table-3. Except for one supermarket, LAMP_SS_-positive samples were found at all sources investigated in this study. The samples collected from 10 of the 20 sources (50%) additionally had positive results for LAMP_SS2_. All samples obtained from supermarkets were LAMP_SS2_ negative, while the samples collected from 6 markets (75%) and 4 butcher shops (50%) were LAMP_SS2_ positive.

**Table-3:** Sources of samples used in this study and LAMP results.

Sample source	Number of sample source	Number of sources containing LAMP_SS_-positive sample (%)	Number of sources containing LAMP_SS2_-positive sample (%)
Market	8	8 (100)	6 (75)
Butcher shop	8	8 (100)	4 (50)
Supermarket	4	3 (75)	0 (0)
Total	20	19 (95)	10 (50)

LAMP=Loop-mediated isothermal amplification

## Discussion

Among *S. suis* infections reported in humans worldwide, >90% of cases have occurred in Asian countries, particularly in Vietnam and Thailand [18]. Since the major cause of *S. suis* human infection in this area is the consumption of a raw pork dish, the microbial food safety hazard associated with raw meat has been raised as a matter of great concern. Therefore, in this study, we investigated the contamination by *S. suis* and *S. suis* serotype 2 or 1/2 in raw pork and edible pig organs sold at different sources in central Thailand using LAMP techniques. Our results showed high contamination rates by *S. suis* in pork and pig organs. In addition, contamination by *S. suis* serotype 2 or 1/2 was found in the samples purchased from markets and butcher shops. *S. suis* and *S. suis* serotype 2 or 1/2 contamination rates in this study were much higher than those reported in a previous study [15], in which the contamination rates of *S. suis* and *S. suis* serotype 2 or 1/2 in raw pork meat samples in Japan were 26.4% and 4.87%, respectively.

In addition, they found that the rate of contamination in pig organs was higher than in pork. Likewise, contamination by *S. suis* was reported in the tongue, tonsil, bone, and tail, but not in pork meat collected from wet markets in Hong Kong [19]. On the contrary, the contamination rates of *S. suis* and *S. suis* serotype 2 or 1/2, in our study, were relatively high in both the pig organs (lung and heart) and meat (sliced pork and minced pork) samples. Although *S. suis* can be found in the tonsil, nasal cavities, and reproductive and digestive tracts of healthy pigs, this bacterium should not be found in sterile sites such as muscle, blood, heart, lung, and liver of a healthy pig [20]. Our findings indicated the possibility of the cross-contamination between visceral organs and pork meat which could occur through slaughtering, meat cutting, meat handling, and further processing [21].

Although many attempts were made, we failed to isolate *S. suis* from the LAMP-positive samples. Since one of the main advantages of LAMP over traditional culture techniques is its sensitivity, it could be suggested that the number of vegetative *S. suis* cells may be too low to compete with the other non-fastidious bacteria contaminating the same sample. Thus, the presence of viable *S. suis* could not be detected using bacterial cultivation. Similarly, Cheung *et al*. [22] revealed that it was difficult to isolate *S. suis* from raw pork meat samples due to the low levels of live *S. suis* cells coexisting with many other microorganisms. However, the retrieval of *S. suis* isolate is required for other important information including virulence and antibiotic resistance traits. Accordingly, it is necessary to further investigate the characteristics of *S. suis* isolates contaminated in raw pork for a better approach to the food safety risk analysis.

Contamination by *S. suis* in raw pork meat was found in all markets and butcher shops examined in this study. Moreover, the samples contaminated with *S. suis* serotype 2 or 1/2 were obtained from 6 of the 8 markets (75%) and 4 of the 8 butcher shops (50%), but no contaminated samples were obtained from the supermarkets. These results emphasized the problem of sanitation and hygienic practices in local retail shops and markets in Thailand. We noticed that the meat handlers in the market stalls worked without gloves and they usually used the same utensils, such as a chopping board and knife, for all meat types. Due to the high contamination rate, the consumers, as well as people who were in close contact with raw pork and pig organs, have a high risk of *S. suis* infection. The achievements from a food safety campaign on the control of *S. suis* infection in Northern Thailand have been described [23]. Therefore, food safety education and public health intervention are crucial to effectively prevent and control the infection of this zoonotic pathogen throughout high-risk areas.

## Conclusion

Using LAMP techniques, high rate contamination of *S. suis* was found in raw pork and edible pig organs sold at different sources in central Thailand. Contamination by *S. suis* serotype 2 or 1/2 was additionally found in samples purchased from markets and butcher shops. It is suggested that the cross-contamination between visceral organs and pork meat possibly occurred during slaughtering, meat cutting, meat handling, and further processing. Therefore, food safety education is necessary to prevent *S. suis* infection.

## Authors’ Contributions

NB collected samples, performed experiments, and wrote the manuscript. SK, DK, and KS provided technical help during the experiments. NM conceived the work, designed the experiments, collected samples, and revised the manuscript critically for important intellectual content. All authors have read and approved the final manuscript.

## References

[1] Lun Z.R, Wang Q.P, Chen X.G, Li A.X, Zhu X.Q (2007). *Streptococcus suis*:An emerging zoonotic pathogen. Lancet Infect. Dis.

[2] Goyette-Desjardins G, Auger J.P, Xu J, Segura M, Gottschalk M (2014). *Streptococcus suis* an important pig pathogen and emerging zoonotic agent-an update on the worldwide distribution based on serotyping and sequence typing. Emerg. Microbes. Infect.

[3] Huong V.T.L, Hoa N.T, Horby P, Bryant J.E, Kinh N.V, Toan T.K, Wertheim H.F.L (2014). Raw pig blood consumption and potential risk for *Streptococcus suis* infection, Vietnam. Emerg. Infect. Dis.

[4] Kerdsin A, Dejsirilert S, Sawanpanyalert P, Boonnark A, Noithachang W, Sriyakum D, Simkum S, Chokngam S, Gottschalk M, Akeda Y, Oishi K (2011). Sepsis and spontaneous bacterial peritonitis in Thailand. Lancet.

[5] Gottschalk M, Segura M (2000). The pathogenesis of meningitis caused by *Streptococcus suis*:The unresolved questions. Vet. Microbiol.

[6] Madsen L.W, Svensmark B, Elvestad K, Aalbaek B, Jensen H.E (2002). *Streptococcus suis* serotype 2 infection in pigs:New diagnostic and pathogenetic aspects. J. Comp. Pathol.

[7] Kerdsin A, Dejsirilert S, Puangpatra P, Sripakdee S, Chumla K, Boonkerd N, Polwichai P, Tanimura S, Takeuchi D, Nakayama T, Nakamura S, Akeda Y, Gottschalk M, Sawanpanyalert P, Oishi K (2011). Genotypic profile of *Streptococcus suis* serotype 2 and clinical features of infection in humans, Thailand. Emerg. Infect. Dis.

[8] Kerdsin A, Hatrongjit R, Gottschalk M, Takeuchi D, Hamada S, Akeda Y, Oishi K (2017). Emergence of *Streptococcus suis* serotype 9 infection in humans. J. Microbiol. Immunol.

[9] Nghia H.D.T, Ngo T.H, Le D.L, Campbell J, To S.D, Chau N.V.V, Mai N.T.H, Tran T.H, Spratt B, Farrar J, Schultsz C (2008). Human case of *Streptococcus suis* serotype 16 infection. Emerg. Infect. Dis.

[10] Takeuchi D, Kerdsin A, Pienpringam A, Loetthong P, Samerchea S, Luangsuk P, Khamisara K, Wongwan N, Areeratana P, Chiranairadul P, Lertchayanti S, Petcharat S, Yowang A, Chaiwongsaen P, Nakayama T, Akeda Y, Hamada S, Sawanpanyalert P, Dejsirilert S, Oishi K (2012). Population-based study of *Streptococcus suis* infection in humans in Phayao province in Northern Thailand. PLoS One.

[11] Taniyama D, Sakurai M, Sakai T, Kikuchi T, Takahashi T (2016). Human case of bacteremia due to *Streptococcus suis* serotype 5 in Japan:The first report and literature review. ID Cases.

[12] Wertheim H.F.L, Nghia H.D.T, Taylor W, Schultsz C (2009). *Streptococcus suis*:An emerging human pathogen. Clin. Infect. Dis.

[13] Meekhanon N, Kaewmongkol S, Phimpraphai W, Okura M, Osaki M, Sekizaki T, Takamatsu D (2017). Potentially hazardous *Streptococcus suis* strains latent in asymptomatic pigs in a major swine production area of Thailand. J. Med. Microbiol.

[14] Wang K, Zhang W, Li X, Lu C, Chen J, Fan W, Huang B (2013). Characterization of *Streptococcus suis* isolates from slaughter swine. Curr. Microbiol.

[15] Arai S, Tohya M, Yamada R, Osawa R, Nomoto R, Kawamura Y, Sekizaki T (2015). Development of loop-mediated isothermal amplification to detect *Streptococcus suis* and its application to retail pork meat in Japan. Int. J. Food Microbiol.

[16] Zhang J, Zhu J, Ren H, Zhu S, Zhao P, Zhang F, Lv H, Hu D, Hao L, Geng M, Gong X, Pan X, Wang C, Qi Z (2013). Rapid visual detection of highly pathogenic *Streptococcus suis* serotype 2 isolates by use of loop-mediated isothermal amplification. J. Clin. Microbiol.

[17] Slater J.D, Allen A.G, May J.P, Bolitho S, Lindsay H, Maskell D.J (2003). Mutagenesis of *Streptococcus equi* and *Streptococcus suis* by transposon Tn *917*. Vet. Microbiol.

[18] Dutkiewicz J, Sroka J, Zajac V, Wasinski B, Cisak E, Sawczyn A, Kloc A, Wojcik-Fatla A (2017). *Streptococcus suis*:A re-emerging pathogen associated with occupational exposure to pigs or pork products. Part I - Epidemiology. Ann. Agric. Environ. Med.

[19] Ip M, Fung K.S, Chi F, Cheuk E.S, Chau S.S, Wong B.W, Lui S, Hui M, Lai R.W, Chan P.K (2007). *Streptococcus suis* in Hong Kong. Diagn. Microbiol. Infect. Dis.

[20] Feng Y, Zhang H, Wu Z, Wang S, Cao M, Hu D, Wang C (2014). *Streptococcus suis* infection:An emerging/reemerging challenge of bacterial infectious diseases?. Virulence.

[21] Adesokan H.K, Raji A.O.Q (2014). Safe meat-handling knowledge, attitudes and practices of private and government meat processing plants'workers:Implications for future policy. J. Prev. Med. Hyg.

[22] Cheung P.Y, Lo K.L, Cheung T.T, Yeung W.H, Leung P.H, Kam K.M (2008). *Streptococcus suis* in retail markets:How prevalent is it in raw pork?. Int. J. Food Microbiol.

[23] Takeuchi D, Kerdsin A, Akeda Y, Chiranairadul P, Loetthong P, Tanburawong N, Areeratana P, Puangmali P, Khamisara K, Pinyo W, Anukul R, Samerchea S, Lekhalula P, Nakayama T, Yamamoto K, Hirose M, Hamada S, Dejsirilert S, Oishi K (2017). Impact of a food safety campaign on *Streptococcus suis* infection in humans in Thailand. Am. J. Trop. Med. Hyg.

